# Impact of attitudes and beliefs on antiretroviral treatment adherence intention among HIV-positive pregnant and breastfeeding women in Zambia

**DOI:** 10.1186/s12889-020-09505-8

**Published:** 2020-09-16

**Authors:** Jerry John Nutor, Jaime C. Slaughter-Acey, Shannon P. Marquez, Rose Ann DiMaria-Ghalili, Florence Momplaisir, Kelechi Elizabeth Oladimeji, Loretta S. Jemmott

**Affiliations:** 1 Family Health Care Nursing Department, School of Nursing, University of California, 2 Koret Way, Suite N431G, San Francisco, CA 94143-0608 USA; 2 Division of Epidemiology and Community Health, University of Minnesota, Minneapolis, MN 55454 USA; 3 Undergraduate Global Engagement, Columbia University, New York City, NY 110027 USA; 4 College of Nursing and Health Professions, Drexel University, Philadelphia, PA 19104 USA; 5 Department of Medicine, Hospital of University of the Pennsylvania, Philadelphia, PA 19102 USA; 6 Department of Public Health, Faculty of Health Sciences, University of Fort Hare, Alice, Eastern Cape South Africa

**Keywords:** Theory of planned behavior, Rural, Pre-natal, Mother-to-child transmission, adherence intention, antiretroviral treatment

## Abstract

**Objective:**

The aim of this study was to investigate if attitudes or behavioral beliefs about antiretroviral therapy (ART) influence ART adherence intention among pregnant and breastfeeding women in Zambia.

**Methods:**

We recruited 150 HIV-positive women receiving ART in urban (Lusaka) and rural (Sinazongwe) districts of Zambia. Generalized modified Poisson regression models were used to assess the extent to which adherence intention was influenced by attitude toward ART or behavioral beliefs about ART.

**Results:**

Intention to adhere to ART differed significantly by income, knowledge about HIV transmission, attitudes, and behavioral beliefs (all Ps < .05). In addition, strong intention to adhere to ART differed by urban (69%) and rural (31%) place of residence (*P* ≤ .01). In adjusted models, women in the weak adherence intention group were more likely to be older, have less knowledge about HIV transmission, and have a more negative attitude toward ART (PR 0.74; 95% CI 0.67–0.82). Behavioral belief about ART, however, was significant in unadjusted model (PR 0.85; 95% CI 0.76–0.94) but not significant after adjusting for covariates such as age, knowledge of transmission, and district locality.

**Conclusion:**

Compared to behavioral beliefs, attitudes about ART were more influential for intention to adhere. This knowledge will help inform effective and appropriate ART counseling for pregnant and breastfeeding women at different points along their ART time course.

## Introduction

There are approximately 85,000 HIV-positive children living in Zambia [1]. The source of infection is primarily through vertical transmission during pregnancy [2], childbirth [2] or breastfeeding from an HIV-positive mother [3, 4]. Administering antiretroviral (ARV) medication to childbearing women who are HIV-positive is one effective strategy to eliminate vertical transmission; however, it requires adherence to the antiretroviral treatment (ART) [5, 6]. Recent studies indicate low rates of adherence to ART among pregnant and breastfeeding women in sub-Saharan Africa [7–9]. In 2012, the World Health Organization (WHO) introduced treatment guidelines (“Option B+”) to reduce vertical transmission of HIV. Option B+ calls for triple ARV therapy (nevirapine, zidovudine and lamivudine) starting as soon as a woman tests positive for HIV and continuing for life regardless of CD4 count or viral load [10]. Option B+ was standard of care in Zambia until 2015, when the WHO expanded to a “90-90-90: Treatment for All” policy with the goal of diagnosing 90% of all people living with HIV, place 90% of diagnosed people on ART, and achieving 90% viral suppression in treated people by 2020.

After the introduction of Option B+, ARV drug use in pregnant and breastfeeding women in Zambia increased from 65% in 2013 to 80% in 2017 [11–13], yet adherence to treatment has been problematic. Data from a recent study conducted in Zambia among women enrolled in Option B+ indicate that 38% did not adhere to the ARV regimen [7]. Poor adherence to ART is related to factors that can be categorized as: patient-related (e.g., forgetting to take the medication or being too busy) [14], interpersonal (i.e. stigma and discrimination) [15], financial difficulty [14], health system (i.e. inaccessibility of services and relationships with service providers) [9, 16] and drug-related (i.e. side effects) [7, 13].

Poor adherence to ART can lead to reduced drug efficacy and increased risk of vertical transmission of HIV at an individual level, and lead to virologic failure [17], drug resistance [18], and vertical transmission [19] of resistant mutations at regional and global levels [14, 15]. Poor adherence can be intentional or unintentional [20, 21]. Unintentional non-adherence occurs when the patient intends to take the drug but circumstances such as forgetfulness, inadequate access to water or food, poor comprehension, or cost of medication limit adherence [9, 14, 15, 22]. Intentional non-adherence occurs when the patient decides not to take a medication as recommended based on one’s attitudes and beliefs [23, 24]. While unintentional non-adherence is common among patients receiving ART, intentional non-adherence has been increasing in low-resource countries [25–27].

According to the Theory of Planned Behavior (TPB) [21], a woman’s intention to take ART is determined by her attitude toward the behavior (taking ARV drugs as prescribed) and her perceived control over the behavior [20, 21]. Attitudes and beliefs influence motivation to start and continue ART [21, 23] and are influenced by education, religion, and socio-cultural norms [28]. In some low-resourced settings, for example, people may doubt the existence of HIV and therefore hesitate to get tested or initiate ART [29–32]. People who believe ART improves health are more likely to adhere to ART than people who have doubts about the disease itself or effectiveness of ARV drugs [23]. To improve ART adherence and reduce maternal-to-child transmission (MTCT), it is important to have a better understanding of childbearing women’s intention to adhere to ART. As one of the top ten countries in the world with the highest prevalence of HIV, Zambia has over 80% of HIV positive pregnant and breastfeeding women currently receiving ART [12, 33]. However, attitudes and beliefs about ART associated with adherence intention has not been examined in Zambian HIV-positive childbearing women receiving ART.

Among TPB constructs, attitudes and behavioral beliefs receive the least attention in research on intention to adhere to ART among women at risk for transmitting the infection to their children [34, 35]. Therefore, the purpose of this study was to investigate how attitudes and behavioral beliefs about taking ART influence ART adherence intention among pregnant and breastfeeding women in Zambia. We hypothesized that positive attitude towards ART and positive behavioral beliefs about taking ART would predict strong adherence intention to ART.

## Methods

### Design and sample

This cross-sectional study of HIV-positive pregnant and breastfeeding women receiving ART took place in urban Lusaka district with predominantly Nyanja and Bemba ethnic groups, and rural Sinazongwe district with a predominantly Tonga ethnic group. The study sites included three hospitals in Lusaka district, and one hospital and two health centers in Sinazongwe district. The selected hospitals and health centers are the main designated clinics for pre- and post-natal care for women living with HIV in the respective districts. Using convenience sampling, we recruited women who received pre- and post-natal care at clinics in both districts. Women were eligible to participate if they were 18 years or older, on ART for at least 2 months, either pregnant or breastfeeding and not intellectually impaired or critically ill (defined as recent hospitalization). The protocol for this study was approved by Drexel University Institutional Review Board and two local institutional review boards: the Eres Converge Institutional Review Board and the Zambia National Health Research Authority.

Pregnant and breastfeeding women diagnosed with HIV infection were contacted in person when they arrived for a clinic visit to obtain their antiretroviral medication or to see their attending midwife or nurse for a regularly scheduled appointment. The midwife/nurse talked to potential participants about the study and women who agreed to learn more about the study were contacted by the trained research assistant. The research assistant explained the purpose, benefits and risks, and confidentiality to potential participants. A copy of the study’s information sheet was given to women who could read. For women who could not read, the research assistant explained the information sheet in a language that the patient understood. After addressing any concerns or questions from potential participants, women who agreed to participate signed a consent form and self-administered questionnaires were distributed. If the participant could not read or write, questions were read in the language they understood best, and responses were recorded on the questionnaire by the research assistant.

### Measures

#### Overview

The outcome and independent predictor variables for this study were adapted from the TPB [20, 21]. In this theory general items are suggested that can be adapted for specific clinical situations. The items underwent various revisions and pilot testing with 10 pregnant and breastfeeding women living with HIV. Results from the pilot test were used to finalize the questionnaire. The items were translated into two local languages (Tonga and Nyanja).

#### Outcome variable: ART adherence intention

In accordance with the TPB [19], adherence intention was measured with four items: (1) “I plan to take my medication consistently as prescribed by the healthcare provider”, (2) “I intend to take my medication consistently as prescribed by the healthcare provider”, (3) “I want to take my medication consistently as prescribed by the healthcare provider”, and (4) “I will take my medication consistently as prescribed by the healthcare provider” [20]. Responses to these items followed a 5-point Likert-type scale (1 = “strongly disagree”, 2 = “disagree”, 3 = “in the middle”, 4 = “agree”, 5 = “strongly agree”). The four responses were summed to create an overall adherence intention score that could range from 4 to 20. Participants were then categorized as having weak adherence intention (score < median of 17) or strong adherence intention (score ≥ 17). Internal consistency reliability was acceptable in our sample (Cronbach α = 0.80).

#### Independent predictor variables

##### Attitude

Attitude toward ART adherence was assessed with an 11-item questionnaire derived from the TPB [20] and included statements such as “Taking ART drugs is easy for me” and “It is tiresome to take MTCT drugs every day” which is reverse-coded [20]. Response options followed a 5-point Likert type scale (1 = “strongly disagree” to 5 = “strongly agree”), resulting in a possible range of 11 to 55. The median score was used to categorize women as having a positive attitude toward ART (≥48) or negative attitude toward ART (< 48). Internal consistency reliability was acceptable in our sample (Cronbach α = 0.77).

##### Behavioral beliefs

To measure behavioral beliefs about taking ART, four items derived from the TPB [20] were created: (1) “I am confident that I will take my ART drugs consistently as prescribed by my health care provider”, (2) “My taking the ART drugs consistently as prescribed by the healthcare provider is useful to me”, (3) “I feel comfortable about talking to my healthcare provider about taking my ART drugs”, and (4) “If I take my ART drugs and experience side effects I will report to the healthcare provider”. Response options were also on a 5-point Likert-type scale (1 = “strongly disagree” to 5 = “strongly agree”), with a possible range from 4 to 20. Higher scores represent more positive beliefs and the median was used to categorize women into negative behavioral beliefs (< 12) or positive behavioral beliefs (≥12). Internal consistency reliability was acceptable in our sample (Cronbach α = 0.76).

### Covariates

#### Knowledge about HIV transmission

To examine knowledge about HIV maternal to child transmission (MTCT)*,* a 13-item questionnaire from Ebuy and colleagues [22] was used with “true” or “false” responses about HIV and MTCT (e.g., “HIV-positive women can reduce the risk of HIV transmission to their babies if they take MTCT drugs”, “Adhering to ARV drugs can reduce the risk of opportunistic infections”). The knowledge score can range from 0 to 13, with higher scores representing a higher level of knowledge. Using the median score of 12, participants were classified as either low (< 12) or high (12–13) knowledge. Internal consistency reliability was acceptable in our sample (Cronbach α = 0.75).

#### Demographic characteristics

he following characteristics were included based on the literature: place of residence (rural or urban) age, marital status (in a relationship living with partner, or in a relationship but not living with partner), education (no formal education or only primary education, secondary, or college and higher), employment (employed, housewife, or informal labor), and monthly household income (< US$200 or ≥ US$200).

### Data analysis

Data entry and cleaning were done in SPSS version 24. Data were analyzed using STATA version 13 (College Station, TX). Descriptive statistics to summarize the variables included means and standard deviations (SD), medians and percentages. To examine adherence intention by the two independent variables, attitudes toward ART and beliefs about ART, bivariate analyses were conducted using modified Poisson regression models to estimate the unadjusted prevalence ratio (PR) and associated 95% confidence interval (CI). In multivariate analyses, models for the two independent variables were built separately using a stepwise approach. Model 1 included attitude or behavioral beliefs, model 2 adjusted for socio-cultural place of residence (urban or rural district), model 3 adjusted for age, marital status, education, monthly household income and occupation in addition to district residence in model 2. Finally, model 4 adjusted for HIV and MTCT knowledge in addition to all the variables in model 3.

#### Sensitivity analysis

Because some researchers have operationalized behavioral intention as a continuous variable [36, 37] rather than categorical as we did in this study, we also conducted a sensitivity analyses to examine potential differences in findings based on how the concept of intention to adhere is operationalized. We performed unadjusted analysis and analyses for model 4 using adherence intention as a continuous variable.

## Results

There were 81 (54%) women from urban district and 69 (46%) from rural district. The average age was 29 years (SD = 6.2) and was similar in both urban and rural districts. The sample was socio-economically diverse; 85% were below the median monthly income of $200 (US equivalent), and 55% had only primary or no formal education. The sample characteristics and results for bivariate analysis are presented in Table 1 by strong and weak intention to adhere groups. Of the 150 women who participated, 77 had strong intention to adhere and 73 were categorized as weak intention to adhere (Table 1). In bivariate analysis, the covariates significantly associated with adherence intention included place of residence (*p* = 0.01), income (*p* = 0.04), and scores for knowledge about HIV and MTCT (*p* < 0.001).

**Table 1:** Adherence Intention to ART in Childbearing Women in Zambia (*N* = 150)

Characteristics	Weak Intention n (%)	Strong Intention n (%)	Chi-square ***P*** value
**Place of residence**			0.01
Urban	28 (38)	53 (69)	
Rural	45 (62)	24 (31)	
**Age, years**			0.15
18–25	11 (15)	23 (30)	
26–30	33 (45)	25 (32)	
31–35	17 (23)	17 (22)	
36–44	12 (16)	12 (16)	
**Living arrangement**			0.35
Married not living with partner	9 (12)	6 (8)	
Married, living with partner	64 (88)	71 (92)	
**Educational status**			0.44
Primary or no formal education	44 (60)	39 (51)	
Secondary	25 (32)	31 (40)	
College/Higher	4 (5)	7 (9)	
**Occupation**			0.35
Employed	16 (22)	25 (32)	
Housewife	36 (49)	33 (43)	
Informal labor	21 (29)	19 (25)	
**Household monthly income**			0.04
< $200	66 (90)	59 (77)	
$200 +	7 (10)	18 (23)	
**Attitude**			< 0.001
Negative	50 (69)	17 (22)	
Positive	23 (31)	60 (78)	
**Behavioral Beliefs**			0.01
Negative	46 (63)	29 (38)	
Positive	27 (37)	48 (62)	
**HIV and MTCT knowledge**			0.01
Low	64 (88)	48 (62)	
High	9 (12)	29 (38)	

### Attitude toward ART

In bivariate analysis, attitude toward ART was significantly (p < 0.001) associated with adherence intention (Table 1). A majority (69%) of women with a negative attitude toward ART were in the weak adherence intention group, and most (78%) women with a positive attitude were in the strong adherence intention group.

In multivariate regression analysis, women with a negative attitude toward ART were less likely to be in the strong adherence intention group in unadjusted analysis (PR 0.73; 95% CI 0.65–0.80) and this association remained consistent and significant in all adjusted analyses (Table 2). For example, in model 4 it can be seen that women with a negative attitude were less likely to be in the strong adherence intention group after adjusting for all other covariates (PR 0.74; 95% CI 0.67–0.82). In addition, residential district remained significantly association with ART adherence intention in all the three adjusted analyses. Age and level of knowledge were also significantly associated with adherence intention in the final adjusted analysis (model 4); indicating that women 26–30 years old were less likely to be in the strong adherence intention group than younger women 18–25 years old (PR 0.85; 95% CI 0.74–0.96), and women with less knowledge were also less likely to be in the strong adherence intention group (PR 0.90; 95% CI 0.80–1.00).

**Table 2:** Multiple Regression Models for Adherence Intention Associated with Attitude Toward ART (*N* = 150)

Characteristics	Model 1	Model 2	Model 3	Model 4
	PR (95% CI)	PR (95% CI)	PR (95% CI)	PR (95% CI)
**Attitude toward ART**				
Positive	Referent	Referent	Referent	Referent
Negative	0.73 (0.65–0.80) **	0.74 (0.68–0.81) **	0.73 (0.66–0.81) **	0.74 (0.67–0.82)**
**District**				
Urban		Referent	Referent	Referent
Rural		0.84 (0.77–0.92) **	0.85 (0.76–0.95)**	0.86 (0.77–0.96)*
**Age**				
18–25			Referent	Referent
26–30			0.84 (0.74–0.95)*	0.85 (0.74–0.96)*
31–35			0.88 (0.76–1.02)	0.90 (0.77–1.04)
36–44			0.93 (0.80–1.09)	0.94 (0.80–1.10)
**Living arrangement**				
Married living with partner			Referent	Referent
Married, not living with partner			0.93 (0.79–1.10)	0.95 (0.80–1.12)
**Educational status**				
College/Higher			Referent	Referent
Primary or no formal education			0.94 (0.76–1.16)	0.97 (0.79–1.20)
Secondary			0.92 (0.75–1.12)	0.94 (0.77–1.16)
**Occupation**				
Employed			Referent	Referent
Housewife			0.98 (0.86–1.11)	1.00 (0.87–1.13)
Informal labor			0.98 (0.85–1.14)	1.00 (0.86–1.16)
**Household monthly Income**				
$200+			Referent	Referent
< $200			0.91 (0.78–1.05)	0.92 (0.79–1.07)
**HIV and MTCT knowledge**				
High				Referent
Low				0.90 (0.80–1.00)*

*p value ≤0.05

**p value < 0.01

### Behavioral beliefs about ART

In bivariate analysis, behavioral belief score was significantly associated with adherence intention (*p* < 0.01). A majority (63%) of the women with negative beliefs about ART were in the weak adherence intention group and a similar percentage (62%) of women with positive beliefs were in the strong adherence intention group (Table 1).

The multivariate analyses for behavioral beliefs about ART and adherence intention are presented in Table 3. Although behavioral belief about ART was significantly associated with adherence intention in unadjusted analysis (PR 0.85; 95% CI 0.76–0.94), the association was no longer significant after adjusting for residential district (model 2) or age and other covariates (model 3).

**Table 3:** Multiple Regression Model for Adherence Intention Associated with Behavioral Beliefs About ART (*N* = 150)

Characteristics	Model 1	Model 2	Model 3	Model 4
	PR (95% CI)	PR (95% CI)	PR (95% CI)	PR (95% CI)
**Behavioral beliefs about ART**				
Positive	Referent	Referent	Referent	Referent
Negative	0.85 (0.76–0.94)**	0.93 (0.82–1.09)	0.91 (0.79–1.06)	0.92 (0.80–1.06)
**District**				
Urban		Referent	Referent	Referent
Rural		0.85 (0.74–0.97)*	0.82 (0.72–0.98)*	0.85 (0.73–0.99)*
**Age, years**				
18–25			Referent	Referent
26–30			0.86 (0.74–0.99)*	0.85 (0.73–1.00)*
31–35			0.92 (0.78–1.08)	0.94 (0.80–1.10)
36–44			0.92 (0.77–1.09)	0.93 (0.78–1.11)
**Living arrangement**				
Married, living with a partner			Referent	Referent
Married, not living with partner			0.90 (0.75–1.09)	0.92 (0.77–1.11)
**Educational status**				
Higher/College			Referent	Referent
Primary or no formal education			0.92 (0.73–1.17)	0.97 (0.76–1.23)
Secondary			0.93 (0.74–1.16)	0.96 (0.77–1.21)
**Occupation**				
Employed			Referent	Referent
Housewife			0.90 (0.78–1.04)	0.92 (0.79–1.07)
Informal labor			1.01 (0.86–1.20)	1.02 (0.87–1.22)
**Household monthly income**				
$200 +			Referent	Referent
< $200			0.94 (0.80–1.11)	0.95 (0.81–1.13)
**HIV and MTCT knowledge**				
High				Referent
Low				0.86 (0.76–0.98)*

*p value ≤0.05

**p value ≤0.01

In the final model, residential district, age and knowledge remained significant. Rural women were 15% less likely to be in the strong adherence intention group compared to urban women (PR 0.85; 95% CI 0.73–0.99). Women 26–30 years old were less likely to be in the strong adherence intention group than younger women 18–25 years old (PR 0.85; 95% CI 0.73–1.00), and women with less knowledge were also less likely to be in the strong adherence intention group (PR 0.86; 95% CI 0.76–0.98).

## Discussion

In this study, the TPB was used for the first time as a framework for examining ART adherence intention among HIV-positive childbearing women in Zambia. In bivariate analysis, both attitude toward ART and behavioral beliefs about ART were significantly associated with adherence intention. However, the adjusted models for adherence intention showed a significant association with attitude but not beliefs, and in general, older rural women with less knowledge were less likely to be in the strong adherence group.

According to the TPB, medication adherence is predicted from intention to adhere, which is grounded in one’s attitudes and beliefs [20]. Attitudes and beliefs are inter-related concepts heavily influenced by one’s socio-cultural environment, but after adjusting for residential district and other factors in our sample, it was attitudes rather than beliefs that were associated with intention to adhere to ART for Zambian women enrolled in the Option B+ treatment regimen.

In our sample of women at risk for MTCT of their HIV infection, positive attitudes and beliefs were associated with strong adherence intention. Other researchers have used the TPB to explain health behaviors [38–40]. In fact, results from a meta-analysis of research utilizing the TPB framework indicated that the TPB could be used to predict 39% of the variance in intention and only 27% of the variance in behavior [41]. Additional studies, therefore, are needed to continue this type of research specifically focused on intention to adhere as a predictor of actual ART adherence behavior.

When Chisholm and colleagues [42] used the TPB to predict adherence to immunosuppressant therapy in renal transplant patients, they found discrepancies in the way the TPB model operated and raised questions about using it to study complex behaviors such as adherence to long-term medication regimens. Similar conclusions were reported by Pellino [43] who used the TPB to investigate analgesic use in adults after elective orthopedic surgery. However, in a sample of Latino HIV-positive adults, Vissman and colleagues [44] found that all components of TPB were significant in predicting adherence to highly active ART. Therefore, for research in this area to progress, further effort is necessary to design valid and reliable measures of the TPB components for specific applications, such as predicting adherence behavior.

Despite the fact that not all the components of the TPB were statistically significant in this study, the results are clinically informative, particularly for health care providers who work in more rural settings. Our findings indicate that women with more negative attitudes and behavioral beliefs about ART are less likely to have a strong intention to adhere to ART. These findings have research implications for designing and testing interventions to enhance patient adherence intention and subsequent adherence to ART. Interventions that specifically target women’s negative attitudes and behavioral beliefs about ART should effectively increase adherence to Option B+. Such interventions, however, should be tailored to individual adherence risk according to the TPB components that provide direction in implementing strategies to foster more positive attitudes and beliefs towards Option B+. Such interventions would ultimately improve overall adherence rates with Option B+.

Our findings also have important policy implications, given that attitude and behavioral beliefs about ART influenced adherence intention among childbearing women in both rural and urban settings. Information about HIV and transmission to the infant should be presented to women prior to initiating ART treatment, and women should be given sufficient time to acknowledge the importance of this information for their own health and the health of entire family. In addition, health educators can use our findings to advocate for appropriate educational programs and materials that reach more vulnerable populations, especially in more rural and low-resourced areas.

The results of this study would be particularly helpful to both clinicians and public health officials because these insights about attitudes and beliefs may be useful in improving adherence intention by paying particular attention to rural communities where socio-cultural resources and knowledge about HIV transmission may be less available. By extension, interventions can be developed and tested to specifically address these negative attitudes and beliefs to promote ART adherence.

### Limitations

Although our findings contribute to a more comprehensive understanding of factors that influence women’s adherence intention to improve health and prevent vertical transmission of HIV to their child, this was a cross-sectional study and it is important to note that these associations are not proof of causation. It is also important to note that all measures in the current study were self-reported. Like all self-report data, some responses might be biased toward social desirability, particularly when a survey is administered face-to-face. The cross-sectional design also does not provide evidence about whether women with a stronger intention to adhere to ART had subsequently better adherence to ART compared to women with a weaker intention. However, evidence from a systematic review indicated that intentions are predictive of subsequent adherence to ART [45]. Furthermore, while data were collected from both a rural and urban district in Zambia, findings may not generalize to all women in Zambia or sub-Saharan Africa. The participants in this study were from a rural part of the Southern Province and the urban part of Lusaka Province, which are culturally and ethnically different from other parts of Zambia. It remains unknown whether our findings regarding attitude and perceived beliefs would be observed in childbearing women from other ethnic groups in Zambia. Finally, while vertical transmission is possible for both pregnant and breastfeeding women, we did not compare these two groups and our results may be influenced by maternal events that occur before or after giving birth.

## Conclusions

Findings from this study suggest that the TPB, specifically attitudes more so than beliefs, can help elucidate key dynamics for improving adherence to ART among Zambian childbearing women. As expected, the theoretical constructs of attitudes and behavioral beliefs were related to intention to adhere to ART Although others have used the TPB to guide their research, measures have largely focused only on attitude, perceived behavioral control, or perceived self-efficacy, and have not addressed specific beliefs related to ART intention. In our present study, negative attitudes and behavioral beliefs were identified as potentially important mediators that should be targeted for interventions designed to increase ART adherence. Future research should be designed to extend the generalizability of our findings to Zambian women of different ethnicities. In light of documented barriers to adherence to ART among Zambian women, the TPB provides a potentially useful framework for researchers and clinicians interested in interventions to increase adherence to ART.

## Data Availability

The datasets generated and/or analyzed during the current study are available from the corresponding author on reasonable request.

## Abbreviations

AIDS: Acquired Immune Deficiency Syndrome
ART: Antiretroviral Therapy
ARV: Antiretroviral
MTCT: Mother-to-child transmission
HIV: Human Immunodeficiency Virus
PR: Prevalence ratio
TPB: Theory of Planned Behavior
WASH: Water Hygiene and Sanitation
WHO: World Health Organization

## References

[1] HIV and AIDS [https://www.unicef.org/zambia/5109_8459.html].

[2] Kourtis AP, Bulterys M, Nesheim SR, Lee FK (2001). Understanding the timing of HIV transmission from mother to infant. J Am Med Assoc.

[3] Embree JE, Njenga S, Datta P, Nagelkerke NJ, Ndinya-Achola JO, Mohammed Z, Ramdahin S, Bwayo JJ, Plummer FA (2000). Risk factors for postnatal mother–child transmission of HIV-1. J Acquir Immune Defic Syndr.

[4] Tubiana R, Le Chenadec J, Rouzioux C, Mandelbrot L, Hamrene K, Dollfus C, Faye A, Delaugerre C, Blanche S, Warszawski J (2010). Factors associated with mother-to-child transmission of HIV-1 despite a maternal viral load< 500 copies/ml at delivery: a case-control study nested in the French perinatal cohort (EPF-ANRS CO1). Clin Infect Dis.

[5] Ogundele M, Coulter J (2013). HIV transmission through breastfeeding: problems and prevention.

[6] World Health Organization (2012). Use of Antiretroviral Drugs for Treating Pregnant Women and Preventing HIV Infection in Infants.

[7] Nutor JJ, Slaughter-Acey JC, Marquez S, Opong E (2019). Factors associated with HIV medication adherence in HIV-positive women enrolled in option B+ in Zambia: a cross-sectional survey. Lancet Glob Health.

[8] Nachega JB, Uthman OA, Anderson J, Peltzer K, Wampold S, Cotton MF, Mills EJ, Ho Y-S, Stringer JS, McIntyre JAJA. Adherence to antiretroviral therapy during and after pregnancy in low-, middle and high income countries: a systematic review and meta-analysis. 2012;26(16):2039. 10.1097/QAD.0b013e328359590f PMC5061936 22951634

[9] Hodgson I, Plummer ML, Konopka SN, Colvin CJ, Jonas E, Albertini J, Amzel A, KPJPo F. A systematic review of individual and contextual factors affecting ART initiation, adherence, and retention for HIV-infected pregnant and postpartum women. 2014;9(11):e111421. 10.1371/journal.pone.0111421 PMC4221025 25372479

[10] Malawi’s Option B+ programme is helping to eliminate mother-to-child transmission of HIV [https://www.unicef.org/infobycountry/malawi_70997.html].

[11] Prevention of mother-to-child transmission (PMTCT) [https://www.who.int/gho/hiv/epidemic_response/PMTCT_text/en/].

[12] HIV/AIDS: Data and Statistics [https://www.who.int/hiv/data/en/].

[13] Nutor JJ, Slaughter-Acey JC, Marquez SP, DiMaria-Ghalili RA, Momplaisir F, Jemmott LS. Influence of toilet access on antiretroviral adherence intention among pregnant and breastfeeding women who are HIV-positive and enrolled in option B+: Health Care for Women International; 2020. p. 1–15. 10.1080/07399332.2020.1746791 32238109

[14] Knettel BA, Cichowitz C, Ngocho JS, Knippler ET, Chumba LN, Mmbaga BT, Watt MH. Retention in HIV care during pregnancy and the postpartum period in the option B+ era: systematic review and meta-analysis of studies in Africa: NIH Public Access; 2018. 10.1097/QAI.0000000000001616 PMC5844830 29287029

[15] Kiwanuka G, Kiwanuka N, Muneza F, Nabirye J, Oporia F, Odikro MA, Castelnuovo B, Wanyenze RK (2018). Retention of HIV infected pregnant and breastfeeding women on option B+ in Gomba District, Uganda: a retrospective cohort study. BMC Infect Dis.

[16] Kaplan R, Orrell C, Zwane E, Bekker L-G, RJA W. Loss to follow-up and mortality amongst pregnant women referred to a community clinic for antiretroviral treatment. 2008;22(13):1679. 10.1097/QAD.0b013e32830ebcee PMC2741732 18670232

[17] Koss CA, Natureeba P, Kwarisiima D, Ogena M, Clark TD, Olwoch P, Cohan D, Okiring J, Charlebois ED, MRJJoaids K. Viral suppression and retention in care up to 5 years after initiation of lifelong ART during pregnancy (option B+) in rural Uganda. 2017;74(3):279. 10.1097/QAI.0000000000001228 PMC5303140 27828878

[18] Nachega JB, Sam-Agudu NA, Mofenson LM, Schechter M, Mellors JW (2018). Achieving viral suppression in 90% of people living with HIV on antiretroviral therapy in low-and middle-income countries: progress, challenges, and opportunities. Clin Infect Dis.

[19] Bezabhe WM, Chalmers L, Bereznicki LR, Peterson GMJM. Adherence to antiretroviral therapy and virologic failure: a meta-analysis. 2016;95(15). 10.1097/MD.0000000000003361 PMC4839839 27082595

[20] Ajzen I. From intentions to actions: a theory of planned behavior. In: Action control: Springer; 1985. p. 11–39.

[21] Ajzen I (2002). Perceived behavioral control, self-efficacy, locus of control, and the theory of planned behavior. J Appl Soc Psychol.

[22] Ebuy H, Yebyo H, Alemayehu M (2015). Level of adherence and predictors of adherence to the option B+ PMTCT programme in Tigray, northern Ethiopia. Int J Infect Dis.

[23] Boateng D, Kwapong GD, Agyei-Baffour P (2013). Knowledge, perception about antiretroviral therapy (ART) and prevention of mother-to-child-transmission (PMTCT) and adherence to ART among HIV positive women in the Ashanti region, Ghana: a cross-sectional study. BioMed Centra Women's Health.

[24] Senkomago V, Guwatudde D, Breda M, Khoshnood K (2011). Barriers to antiretroviral adherence in HIV-positive patients receiving free medication in Kayunga, Uganda. J Acquir Immune Defic Syndrome Care.

[25] Mo PK, Mak WW (2009). Intentionality of medication non-adherence among individuals living with HIV/AIDS in Hong Kong. AIDS Care.

[26] Kamuhabwa A, Bakari M. The magnitude of intentional non-adherence to antiretroviral therapy among patients attending HIV care and treatment clinic at Muhimbili National Hospital, Dar es Salaam, Tanzania. Tanzania Med J. 2009;24(2).

[27] Kalichman S, Mathews C, Banas E, Kalichman M (2019). Alcohol-related intentional nonadherence to antiretroviral therapy among people living with HIV, Cape Town, South Africa. AIDS Care.

[28] Jemmott JB, Jemmott LS, Braverman P, Fong G (2005). HIV/STD risk reduction interventions for African American and Latino adolescent girls at an adolescent medicine clinic: a randomized controlled trial. Arch Pediatr Adolesc Med.

[29] Deribew A, Abebe G, Apers L, Jira C, Tesfaye M, Shifa J, Abdisa A, Woldemichael K, Deribie F, Bezabih M (2010). Prejudice and misconceptions about tuberculosis and HIV in rural and urban communities in Ethiopia: a challenge for the TB/HIV control program. BMC Public Health.

[30] Yaya S, Ghose B, Udenigwe O, Shah V, Hudani A, Ekholuenetale M (2018). Knowledge and attitude of HIV/AIDS among women in Nigeria: a cross-sectional study. Eur J Pub Health.

[31] Gazimbi MM, Magadi MA (2019). Individual-and community-level determinants of antenatal hiv testing in Zimbabwe. J Biosoc Sci.

[32] Nutor JJ, Duah HO, Agbadi P, Duodu PA, Gondwe KW (2020). Spatial analysis of factors associated with HIV infection in Malawi: indicators for effective prevention. BMC Public Health.

[33] HIV/AIDS [http://www.who.int/mediacentre/factsheets/fs360/en/].

[34] Senkowski V, Gannon C, Branscum P (2019). Behavior change techniques used in theory of planned behavior physical activity interventions among older adults: a systematic review. J Aging Phys Act.

[35] Saal W, Kagee A (2012). The applicability of the theory of planned behaviour in predicting adherence to ART among a south African sample. J Health Psychol.

[36] Jalilian F, Mirzaei-Alavijeh M, Ahmadpanah M, Mostafaei S, Kargar M, Pirouzeh R, Sadeghi Bahmani D, Brand S. Extension of the Theory of Planned Behavior (TPB) to predict patterns of marijuana use among young Iranian adults. Int J Environ Res Public Health. 2020;17(6). 10.3390/ijerph17061981 PMC7142430 32192209

[37] Zhu G, Qian X, Qi L, Xia C, Ming Y, Zeng Z, Liu Y, Yang Y, Zhang M, Zhang H. The intention to undertake physical activity in pregnant women using the theory of planned behaviour. J Adv Nurs. 2020. 10.1111/jan.14347 32153052

[38] Johnston KL, White KM (2003). Binge-drinking: a test of the role of group norms in the theory of planned behaviour. Psychol Health.

[39] Villarruel AM, Jemmott JB, Jemmott LS, Ronis DL (2004). Predictors of sexual intercourse and condom use intentions among Spanish-dominant Latino youth: a test of the planned behavior theory. Nurs Res.

[40] Jemmott JB, Heeren G, Ngwane Z, Hewitt N, Jemmott L, Shell R, O'leary A (2007). Theory of planned behaviour predictors of intention to use condoms among Xhosa adolescents in South Africa. AIDS Care.

[41] Armitage CJ, Conner M (2001). Efficacy of the theory of planned behaviour: a meta-analytic review. Br J Soc Psychol.

[42] Chisholm MA, Williamson GM, Lance CE, Mulloy LL (2007). Predicting adherence to immunosuppressant therapy: a prospective analysis of the theory of planned behaviour. Nephrol Dialysis Transplant.

[43] Pellino TA (1997). Relationships between patient attitudes, subjective norms, perceived control, and analgesic use following elective orthopedic surgery. Res Nurs Health.

[44] Vissman AT, Hergenrather KC, Rojas G, Langdon SE, Wilkin AM, Rhodes SD (2011). Applying the theory of planned behavior to explore HAART adherence among HIV-positive immigrant Latinos: elicitation interview results. Patient Educ Couns.

[45] Rich A, Brandes K, Mullan B, Hagger MS (2015). Theory of planned behavior and adherence in chronic illness: a meta-analysis. J Behav Med.

